# Novel Approaches to Detect and Treat Biofilms within the Root Canals of Teeth: A Review

**DOI:** 10.3390/antibiotics9030129

**Published:** 2020-03-20

**Authors:** Laurence J. Walsh

**Affiliations:** Faculty of Health and Behavioural Sciences, The University of Queensland School of Dentistry, UQ Oral Health Centre, 288 Herston Road, Herston, QLD 4006, Australia; l.walsh@uq.edu.au

**Keywords:** biofilms, fluorescence detection, continuous chelation, fluid agitation, nanoparticles

## Abstract

Biofilms located within the root canals of teeth are a unique and pressing concern in dentistry and in medical microbiology. These multispecies biofilms, which include fungi as well as bacteria, form in a protected site with low shear stress and low oxygen tension. Systemic antibiotics are of limited value because of the lack of blood flow of the site, and issues with innate and acquired resistance. Physical disruption using hand or rotary powered instruments does not reach all locations in the root canal system where biofilms are present. Alternative strategies including agitated irrigation fluids, continuous chelation, materials with highly alkaline pH, and antimicrobial nanoparticles are being explored to meet the challenge. Detection and quantification of biofilms using fluorescence-based optical methods could provide an indication of successful biofilm removal and an endpoint for physical and chemical treatments.

## 1. Introduction

The progression of dental caries (tooth decay) through the crowns and roots of teeth can result in necrosis of the dental pulp and invasion of oral microorganisms into the root canal system of the tooth. Infection within the root canal system of a tooth is difficult to treat. The multiple species of microorganisms that are present (typically over 20 species of bacteria as well as several fungi) (Table 1) invade dentinal tubules, accessory canals, canal ramifications, apical deltas, fins and transverse anastomoses, where they form dense multispecies biofilms [1,2,3,4,5]. Biofilms form not only within the root canal, but also on the root surface, in the apical region.

**Figure 1:**
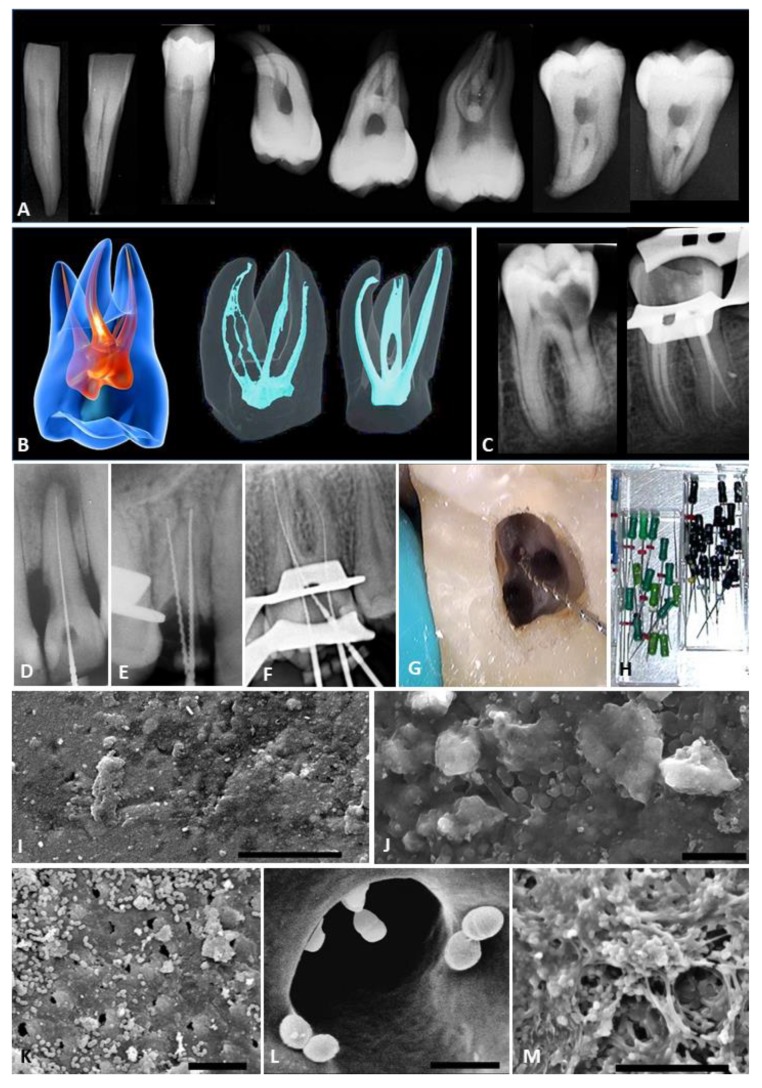
The challenges of traditional endodontic treatment. Panel A shows radiographs (from left to right) of two mandibular incisors, a mandibular premolar, three maxillary molars and two mandibular molars. These are typical examples of internal anatomy of teeth, and show multiple root canals, and complex root curvatures. These root canal systems are difficult to instrument effectively.

**Figure 2:**
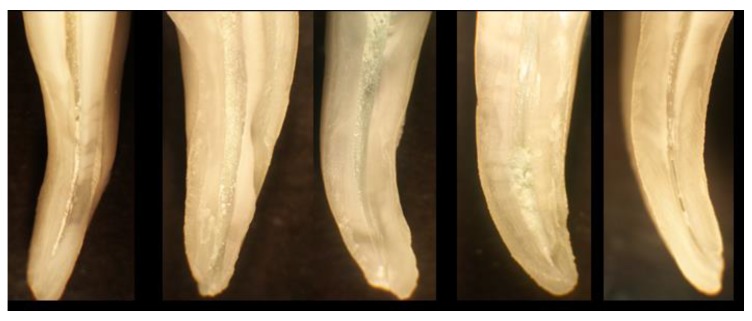
Examples of roots of molar teeth that have been instrumented using hand files, and then sectioned using a diamond saw, to reveal the walls of the root canals. These walls should be smooth and completely free of biofilm, however all these examples show debris and remnants of biofilm (yellow-white granular deposits) along the walls of the root canals.

The primary goal of endodontics (root canal treatment) is to eliminate microorganisms from the root canal system and from the associated dentine of the root, prior to placing a root canal filling to occupy the space [6]. Current approaches to endodontic treatment (Figure 1 and Figure 2) are based on instrumentation, combined with irrigation and medication of the root canal system [7]. Traditional hand and powered endodontic files do not contact all the walls of the root canal [8] and thus, biofilm remnants remain in areas that are difficult to access, such as fins, deltas and lateral canals (Table 2). These persisting microorganisms and their associated products (such as endotoxins) can cause persisting pain, periapical inflammation and bone loss [9,10,11].

## 2. The Challenge of Biofilms in the Root Canal System

Biofilms within the root canal system of teeth exist in a protected environment with a low oxygen tension, a low pH, and little if any flow of fluid [12]. The absence of shear forces distinguishes these biofilms from oral biofilms such as dental plaque on the surfaces of teeth. The structure of these biofilms limits diffusion of active ingredients from medicaments. In addition, the dentine walls of the root canal interact with some medicaments and reduce their effectiveness. Achieving reliable and complete decontamination and disinfection remains a major challenge.

Panel B of Figure 1 shows on the left a simplified “textbook” representation of the root canal system (shown in red) of a maxillary molar tooth. This oversimplifies the actual internal anatomy, as shown by micro-computed tomography, in the center and right images. The micro-CT images are based on data from Reference 66. Panel C shows radiographs of a mandibular molar tooth. The left image is prior to endodontic treatment. Note that the radiograph does not reveal the true complexity of the root canal system. The right radiograph shows the same tooth immediately after endodontic treatment, showing four separate canals that have been filled. Panels D-F are radiographs taken during endodontic treatment of a maxillary incisor with one root canal, a maxillary premolar with two root canals, and a maxillary molar with three root canals. Metallic hand files are present in the canals. These files are used for determining working length as well as for exploration, internal shaping and mechanical debridement. Panel G is a clinical view through an operating microscope showing a file inserted into one of the four canals of a maxillary first molar. Panel H shows hand files used in endodontic treatment. These come in various shapes and profiles, with diameters beginning as small as 8 μm (size ISO 08). Panel I is a scanning electron microscope view of a typical multispecies biofilm left on the walls of the root canal in a protected area that instruments have not reached. The scale bar is 5 μm. Panel J is another SEM view of a typical endodontic biofilm showing individual microorganisms and extracellular polymeric substances. The scale bar is 10 μm. Panel K shows an area of the root canal walls in the middle third of the root canal system where there are layers of individual bacteria, sitting near and within the opening of dentinal tubules. The scale bar is 10 μm. Panel L shows a high power SEM view of the coronal aspect of the root canal system (where the dentinal tubules have the largest diameter). Note the relative size of the bacteria compared to the opening of the dentinal tubule. These bacteria typically penetrate as far as 300 μm into dentinal tubules. Panel M shows a typical multispecies biofilm in the middle third of the root canal system. A diversity of microorganisms are present. The scale bar is 10 μm. 

In terms of treatment endpoints, knowing the point at which a suitable level of biofilm removal has been achieved is a further challenge. In response to depletion of substrates and the accumulation of waste products, some bacteria enter a quiescent phenotypic state with altered gene expression and no active replication. This state makes them no longer susceptible to antibiotics that target the metabolic pathways of bacterial replication. The bacteria are alive, but they cannot readily be detected by culture-based methods [13]. 

Resistance of bacteria in root canal biofilms to antibiotics is a further problem. The extracellular polymeric substances (EPS) that comprise the matrix of these biofilms retards diffusion of antibiotics. When combined with the influence of the change in the growth state of bacteria, this translates into a 1000-1500 times greater resistance to antibiotics than when the same organisms are in the freely dispersed planktonic form. The mixed flora that is present may also contain many organisms that are inherently resistant to the antibiotics being used [14]. 

Subpopulations of some Gram-positive bacteria in a biofilm may enter a phenotypic state with altered gene expression which is akin to spore formation. This has been well documented for *Enterococcus faecalis*. This organism, because of its well-deserved reputation as a difficult target for antibiotic and antimicrobial therapy, has been used as the target for many studies in the endodontic literature [15]. *E. faecalis* is inherently more resistant to antimicrobial drugs than any other clinically important Gram-positive bacteria encountered in dentistry. It is resistant to cephalosporins, clindamycin, and aminoglycosides [16,17]. 

Because of these challenges, a range of novel approaches have been developed for detecting and treating biofilms within the root canals of teeth. Real-time assessment using fluorescence methods is the most promising practical method for detecting biofilms as well as planktonic microorganisms. Methods for enhanced inactivation of endodontic biofilms can be divided into improved antimicrobial irrigation solutions, alkaline medicament pastes, and nanoparticles (Table 3). Each approach is promising, but significant technical challenges remain for the full implementation of these into everyday clinical practice.

## 3. Real-Time Assessment of Endodontic Biofilms Using Fluorescence Methods

Traditional culture-based techniques for detecting microorganisms, such as sampling the root canal system using sterile paper points, are not suitable for use in the everyday clinical practice of endodontics. There is a great diversity of the organisms that are present, both in planktonic form and embedded within biofilms. Both facultative and strict anaerobes will be present, making recovery and later, analysis in the laboratory challenging, time-consuming, and expensive. 

A variation on the paper point sampling approach is to use a fluorescent dye for analysis of the sample taken rather than attempting to culture it. This approach should give greater sensitivity than conventional culture because it will work on organisms that have been recovered from nutritionally deprived, stressed biofilms.

The dye calcein-AM (Calcein acetoxymethyl ester) has been used for detecting vital bacteria recovered from root canals on adsorbent paper points [18]. The dye is incubated with the sample for 5 min and the sample then evaluated using fluorescence and micro-spectroscopy with software-based spectral analysis. When used in a clinical setting, this dye method was found to be superior to traditional culture-based methods, and was more sensitive, being able to detect lower cell numbers. The method requires specialized equipment, which limits its usefulness. A further limitation is that paper point sampling collects fluids by capillary action from the central portion of the root canal system. Using a paper point, it is not possible to recover organisms from protected or inaccessible areas, such as deep within dentinal tubules, or within fins.

These same issues apply when samples are collected for molecular analyses using paper points. Molecular methods, like conventional culture-based methods, require a laboratory, are technically challenging and expensive, and do not provide real-time information. A further issue is that molecular analysis methods that are based on 16S rDNA will provide insight into bacterial diversity, but will not detect fungi [19,20].

To overcome such problems and provide real-time assessment of the presence of microorganisms in the root canal system, fluorescence methods have been developed. The broad objective of these is to provide information that can guide clinical decisions around treatment endpoints, by answering the question, “Are there still microorganisms present in the root canal?” If the answer is negative, this provides an endpoint for treatment, and will prevent excessive instrumentation of the tooth.

### 3.1. Fluorescence Using Ultraviolet Light or Visible Violet Light

There are several variations on the fluorescence diagnostic approach. The first group of these methods uses long wavelength ultraviolet light or visible violet light (e.g., 385–405 nm wavelength), such as from a suitable diode laser or LED. The light is used in combination with a suitable fluorescent dye [21], or without a dye, exploiting intrinsic fluorescence properties of tooth structure and microbial deposits. Both long wavelength ultraviolet light and visible violet light elicit strong visible red emissions from bacterial biofilms, because of the presence of endogenous fluorophores, such as porphyrins that are involved in iron metabolism. “Black-pigmented” organisms give strong visible red emissions when exposed to ultraviolet or visible violet light because of their high porphyrin content. The same effects occurs in mature dental plaque biofilms on the surfaces of teeth and in dental caries [22,23,24]. There are also green emissions from the collagen component of dentine. Analyzing these emissions using quantitative fluorescence spectroscopy can help detect microbial deposits and pulpal soft tissue remnants within the root canal system [25]. Quantitative fluorescence spectroscopy using ultraviolet light may also detect changes in the dentine collagen that are due to proteolysis. The major limitations of using long wavelength ultraviolet light are the design of a suitable optical probe and the need for sophisticated spectral analysis equipment.

The choice of the light wavelength used for fluorescence excitation has other practical implications. Light in the long wavelength ultraviolet wavelength range has a higher photon energy than visible light and thus, gives a greater quantum yield when used for fluorescence excitation; however, it scatters strongly, and has poor penetration into tooth structure [26]. 

### 3.2. Fluorescence Using Visible Red Light

Visible red light also excites bacterial porphyrins, causing emission of near infrared light. Both visible red and near infrared light transmit well through tooth structure [27,28,29]. This high degree of penetration for both wavelengths allows detection of bacteria within tooth structure, including within dentine tubules. Using visible red light (655 nm), an existing low cost laser fluorescence device, the DIAGNOdent (KaVo, Biberach, Germany) has been used in a proof-of-concept study, with a rigid sapphire tip placed into the pulp chamber and the coronal third of the root canal system in extracted teeth. The fluorescence properties of bacterial cultures, mono-species biofilms grown in root canals, pulpal soft tissues, and sound dentin have been examined with the DIAGNOdent, together with 50 extracted teeth with known endodontic pathology [30]. Both sound dentin and healthy pulpal soft tissue gave an average fluorescence reading of 5 (on a scale of 100). This is below the threshold reading of 7 for healthy dental soft tissues and healthy tooth surfaces that are free of microbial biofilms [31,32]. High fluorescence readings were recorded in the root canals and pulp chambers of extracted teeth with radiographic evidence of periapical pathology and scanning electron microscopy evidence of bacterial infection and polymicrobial biofilms. When biofilms were created in the root canals under laboratory conditions, as these biofilms matured, there was a progressive increase in fluorescence over time. When teeth with mono-species or multi-species biofilms in the root canal underwent endodontic treatment, the fluorescence readings reduced to below the "healthy" threshold. 

The positive results from investigations using fluorescence assessment of the status of the pulp chamber and root canal system using a miniature rigid fiber-optic tip then led to studies using flexible tips that could reach into the middle and apical thirds of the root canal to detect biofilms and planktonic microorganisms. Penetration of flexible optical fibers was tested on sectioned extracted teeth. Fluorescence recordings were made ex vivo in the canals of extracted teeth with known periapical pathology. The fibers were able to reach the apical third of the root canal, unless the canals had very pronounced distal curvatures (i.e., greater than 15 degrees). Fibers with conical modified ends penetrated better than those with plain ends. In keeping with previous studies, fluorescence readings were significantly higher in infected canals with biofilms, and these readings returned to below the “healthy” threshold once the root canals were instrumented to the correct working length [33]. It was therefore concluded that fluorescence analysis of root canals using flexible optical fiber probes has the potential for real-time assessment of the microbial status of the root canal system in clinical practice. 

Later work developed modifications to the miniature flexible optical fibers to increased their lateral emission and lateral light collecting properties, and thus maximize the detection capabilities of this method [34,35]. A particular honeycomb surface configuration with grating-like properties was micro-patterned onto a conical tip. This was found to give considerable improvements in the lateral emission and collection of light. Using this tip with visible red light that passes readily through dentine allowed detection of bacteria located within dentine tubules. Such methods could reveal areas of the root canal system that had not yet been instrumented adequately and they could also provide an endpoint to treatment of a particular canal in a tooth with multiple canals.

## 4. Improved Antimicrobial Irrigant Solutions

Biofilms in the root canal are difficult to reach with round endodontic files [36]; hence, files with novel shapes that can move in eccentric or oval paths have been developed. These designs are more effective in canals with oval cross-sections than circular files [37,38]. Nevertheless, any given rotary instrumentation system needs to be combined with a suitable antimicrobial irrigant solution, and a suitable delivery or agitation technique for that solution, to achieve chemo-mechanical debridement of the root canal system.

The viscosity and surface tension of the antimicrobial irrigant used with endodontic files influences how well that irrigant touches the biofilm on the walls of the root canal and the sides of the files being used. Including surfactants helps overcome problems such as vapour locks and helps the irrigation fluid enter into fins and lateral canals and other areas that are more difficult to access. 

As partners to physical debridement, most current clinical protocols involve copious irrigation with 2.5–6% solutions of sodium hypochlorite (NaOCl). These formulations of NaOCl also contain sodium chloride, sodium hydroxide (as a pH modifier) and one or more surfactants. A major point of difference between endodontic NaOCl irrigant solutions and domestic bleach is that the endodontic formulations are more alkaline (with a pH from 10 to 12), and are better able to dissolve vital and non-vital soft tissues because of the combined actions of the hypochlorite anion and hydroxyl ions [39]. 

While NaOCl irrigation solutions have antimicrobial actions against both bacteria and fungi, some highly resistant organisms, particularly *E. faecalis*, require longer exposure times of up to 5 min for inactivation when in the biofilm state [40]. Bacteria that are lodged deep within dentinal tubules will be protected from contact with NaOCl, and the high pH of this irrigant solution will be buffered by the adjacent dentine [41]. Methods to enhance the action of NaOCl against root canal biofilms include adding in various detergents such as cetrimide or benzalkonium chloride to lower the surface tension and enhance penetration of the solution into dentinal tubules [42,43]. Other ways to enhance the action of NaOCl include adding in calcium hydroxide, which enhances its antimicrobial actions and reduces the levels of bacterial endotoxins [44], and physical activation of the solution, by using ultrasonic instruments or pulsed middle infrared lasers (such as Er:YAG or Er,Cr:YSGG lasers). Ultrasonic agitation with piezoelectric ultrasonic instruments requires a moving tip, and creates random cavitation events, while laser agitation uses a stationary tip and creates orchestrated cavitation events. This explains why lasers are superior to ultrasonic instruments for agitation.

With some agitation protocols, biofilm may still remain in the apical root canal third, or bacteria persist in infected dentinal tubules [45]. To address such issues, novel laser protocols and optical fiber tips have been developed to enhance cavitation and the movement of irrigation fluids in the root canal system. These remove debris as well as organic residues created by instruments (termed the smear layer) [46,47]. This enhanced removal effect occurs because of greater physical contact of the irrigant solution with the biofilm on the root canal walls, as well as a mild elevation in the temperature of the irrigant solution. The latter does not, however, cause thermal stress to cells of the periodontal ligament on the root surface of the tooth that is being treated.

Traditionally, NaOCl irrigation is followed by irrigation with a chelating agent (such as EDTA), to ensure removal of smear layer. There are new protocols termed “continuous chelation” where both the chelating agent and the NaOCl are present at the same time (i.e., mixed together). This is not possible using EDTA since it interacts chemically with NaOCl, consuming the free available chlorine, and rendering the mixture inactive [48]. Recent research has shown the value of certain non-nitrogen-containing bisphosphonates, such as clodronate, for use in continuous chelation, since they do not react with NaOCl, and give stable mixtures with excellent smear layer removal capabilities as well as powerful antimicrobial actions [49,50]. The continuous chelation approach may be able to reduce time and complexity, as well as improve the quality of debridement of the canal by its more potent actions on biofilms and smear layer. A reduction in bacterial counts is certainly desirable, but may not be the only factor to be considered [51]. 

Consideration must also be given to the impact of the irrigant fluid when it is extruded beyond the confines of the root canal system. Extrusion of NaOCl, even at very small volumes, causes pain, swelling and inflammation [52]. Clodronate exerts anti-inflammatory and analgesic actions, as well as modulating bone metabolism. These anti-inflammatory and analgesic effects have been exploited in the management of osteoarthritis [53]. Based on the positive clinical findings from such clinical applications, as a new use for an old drug, continuous chelation with clodronate combined with NaOCl would be expected to reduce the prevalence of post-endodontic pain and thus, the need for analgesic medications following root canal treatment. Post-endodontic pain is a common clinical problem, and may occur in as many as 58% of patients following root canal treatment [54]. Including clodronate, which maintains the potency of NaOCl, is a better option than simply reducing the concentration of NaOCl, as this will reduce its antimicrobial actions [55].

## 5. Antimicrobial Medicaments

When considering the diversity of the species of bacteria and fungi that may be present in the biofilms that form in an infected root canal system (Table 1), the medicaments applied must have broad spectrum activity. *E. faecalis* must be kept at a pH level of over 11 to be inactivated, yet this pH is not achieved in the root canal when water-based calcium hydroxide pastes are used, because of pH buffering by dentine proteins [56,57].

One solution to this challenge is to deploy calcium hydroxide using a paste with a specific non-water polymer vehicle that enhances the release of calcium and hydroxyl ions, achieving a higher pH to inactivate both bacteria as well as fungi [58]. Despite elevating the pH, such materials do not weaken teeth, even when placed into the root canal for prolonged periods [59,60].

Other than calcium hydroxide, there is also growing interest in non-antibiotic antimicrobial agents which can penetrate biofilms for possible inclusion in endodontic medicaments, including plant-derived phenolics, and nanoparticles such as chitosan which can inactivate both fungi and bacteria [61].

Both silver nanoparticles [62,63,64] and biomimetic iron oxide nanoparticles have been shown to impair biofilm formation and to prevent dentinal tubule infection by *E. faecalis* [65]. This approach should work even in teeth with complex canals [66]. Silver nanoparticles are attractive because they can be biosynthesized by endophytic fungi such as *Fusarium semitectum* with high efficiency and low cost. They could be added to root canal irrigant solutions, or to intra-canal medicament pastes.

A summary of implementation issues with new methods to deal with endodontic biofilms is presented in Table 3.

## 6. Conclusions

Endodontic biofilms are well protected within the root canals of teeth and are difficult to access. Their effective elimination is essential for long-term clinical success and remains a major goal for endodontic research. Knowing that biofilms are present in the root canal systems is essential information for clinical practice, since it drives the need for additional treatments, whereas the absence of microorganisms is an endpoint for treatment. 

Using the endogenous fluorescence of porphyrins and other bacterial metabolites for real-time detection and analysis of the presence and extent of these biofilms is a practical approach, with penetrating visible red light as the preferred excitation source. It is likely that an optimal approach for biofilm removal will require the combination of several of the technologies discussed in this review, including improved methods for mechanical removal of biofilms using instrumentation combined with irrigation fluids, as well as enhanced chemical treatments and improved biocides that can inactivate microorganisms. The use of antimicrobial nanoparticles is an attractive avenue for further work, particularly as these could readily be added to existing irrigation fluids. Because endodontic infection is mediated by biofilms, the approaches taken must deal with the challenges posed by the quiescent metabolic state of microorganisms in the biofilm, and address the problem of growing microbial resistance to antibiotics by exploiting other methods of treatment to their fullest extent. As a result, there will be a need for decreased reliance on traditional antibiotics, and greater utilization of antimicrobial strategies for which the development of resistance is unlikely to occur. 

## Figures and Tables

**Table 1:** The complex composition of endodontic biofilms.

Bacteria—summary Over 400 different bacterial species have been identified in the root canal of teeth Endodontic biofilms typically contain around 20 species, but can have many as 30 or more species of bacteria The most frequent bacteria in endodontic biofilms belong to the phyla *Firmicutes, Proteobacteria, Spirochaetes, Bacteroidetes*, and *Actinobacteria*
Gram-positive bacteria Common streptococci include *Streptococcus intermedius, S. constellatus* and *S. mutans*, and other facultative or microaerophilic streptococci Common enterococci include *Enterococcus faecalis* Gram-positive anaerobes include species belonging to the genera *Peptostreptococcus*, *Eubacterium*, and *Pseudoramibacter*
Gram-negative bacteria Gram-negative anaerobic bacteria include species belonging to the genera *Fusobacterium*, *Porphyromonas*, *Prevotella*, and *Campylobacter*. Commonly found Gram-negative bacteria include *Tannerella forsythia*, *Porphyromonas gingivalis, P. endodontalis, Prevotella* *intermedia, P. nigrescens*, *Fusobacterium periodonticum*, *F. nucleatum*, and *Eikenella corrodens* Spirochaetes (treponemes) include *Treponema denticola, T. socranskii, T. maltophilum, T. lecithinolyticum, T. vincentii, T. pectinovorum, T. amylovorum*, and *T. medium*
Archaea, such as *Methanobrevibacter oralis* and *M. filiformis*.
Fungi, including *Candida albicans*

**Table 2:** Traditional methods used for the treatment of endodontic biofilms.

Method	Major Limitations
Physical debridement	
Hand endodontic files	Non-contact with walls of the root canal; instrument breakage
Rotary endodontic files	Limited contact with walls; excessive root structure removal
Ultrasonic endodontic files	Limited activation of irrigant fluid; apical fluid extrusion
Irrigant solutions	
Sodium hypochlorite	Chemical irritancy; chemical instability; instrument corrosion
Hydrogen peroxide	Chemical irritancy; interactions with other irrigant solutions
Chlorhexidine	Limited spectrum of activity; chemical degradation
EDTA	No antimicrobial actions; inactivates sodium hypochlorite
Medicament pastes	
Calcium hydroxide	Limited alkaline pH for aqueous preparations
Phenolic compounds	Limited spectrum of activity; chemical irritancy
Tetracyclines	Staining of roots from first generation tetracyclines
Clindamycin	Inherent resistance of *E. faecalis*; adverse reactions

**Table 3:** New methods of addressing the challenges of endodontic biofilms.

Technology	Issues to Be Resolved
Fluorescence detection	Quenching of fluorescence emissions (e.g., by oxidants) Flexibility of optical fibers that penetrate the root canal system
Laser fluid agitation	Laser pulse patterns to optimize fluid agitation fluid extrusion
Sodium hypochlorite	Stabilizers and surfactants to optimize performance
Clodronate	Extent of anti-inflammatory actions
Phytochemicals	Spectrum of activity; irritancy and toxicity
Chitosan	Particle size; consistency of compositions
Metal nanoparticles	Penetration into biofilms; toxicity to the host
